# BioCreative III interactive task: an overview

**DOI:** 10.1186/1471-2105-12-S8-S4

**Published:** 2011-10-03

**Authors:** Cecilia N Arighi, Phoebe M Roberts, Shashank Agarwal, Sanmitra Bhattacharya, Gianni Cesareni, Andrew Chatr-aryamontri, Simon Clematide, Pascale Gaudet, Michelle Gwinn Giglio, Ian Harrow, Eva Huala, Martin Krallinger, Ulf Leser, Donghui Li, Feifan Liu, Zhiyong Lu, Lois J Maltais, Naoaki Okazaki, Livia Perfetto, Fabio Rinaldi, Rune Sætre, David Salgado, Padmini Srinivasan, Philippe E Thomas, Luca Toldo, Lynette Hirschman, Cathy H Wu

**Affiliations:** 1 Center for Bioinformatics and Computational Biology, University of Delaware, Newark, DE, USA; 2 Pfizer Research Technology Center, Cambridge, Massachusetts, USA; 3 Medical Informatics, University of Wisconsin-Milwaukee, Milwaukee, Wisconsin, USA; 4 Department of Computer Science, The University of Iowa, Iowa City, Iowa, USA; 5 University of Rome Tor Vergata, Italy; 6 IRCCS Fondazione Santa Lucia, Italy; 7 Wellcome Trust Centre for Cell Biology, University of Edinburgh, UK; 8 Institute of Computational Linguistics, University of Zurich, Zurich, Switzerland; 9 CALIPHO group, Swiss Institutes of Bioinformatics, Geneva, Switzerland; 10 dictyBase, NIBIC, Northwestern University, Chicago, IL, USA; 11 University of Maryland, Baltimore, MD, USA; 12 TAIR, Carnegie Institution for Science, Washington, DC, USA; 13 Structural and Computational Biology Group, Spanish National Cancer Research Centre (CNIO), Madrid, Spain; 14 Humboldt-Universität zu Berlin, Unter den Linden 6, 10099 Berlin, Germany; 15 National Center for Biotechnology Information (NCBI), Bethesda, MD, USA; 16 MGI, The Jackson Laboratory, Bar Harbor, ME, USA; 17 Department of Computer Science, University of Tokyo, Japan; 18 Department of Computer and Information Science, NTNU, Trondheim, Norway; 19 Australian Regenerative Medicine Institute, Monash University, Melbourne, Victoria, Australia; 20 Developmental Biology Institute of Marseille Luminy (IBDML), Université de la Méditerranée, Campus de Luminy, Marseille, France; 21 Merck KGaA, Darmstadt, Germany; 22 Information Technology Center, The MITRE Corporation, Bedford, MA, USA

## Abstract

**Background:**

The BioCreative challenge evaluation is a community-wide effort for evaluating text mining and information extraction systems applied to the biological domain. The biocurator community, as an active user of biomedical literature, provides a diverse and engaged end user group for text mining tools. Earlier BioCreative challenges involved many text mining teams in developing basic capabilities relevant to biological curation, but they did not address the issues of system usage, insertion into the workflow and adoption by curators. Thus in BioCreative III (BC-III), the InterActive Task (IAT) was introduced to address the utility and usability of text mining tools for real-life biocuration tasks. To support the aims of the IAT in BC-III, involvement of both developers and end users was solicited, and the development of a user interface to address the tasks interactively was requested.

**Results:**

A User Advisory Group (UAG) actively participated in the IAT design and assessment. The task focused on gene normalization (identifying gene mentions in the article and linking these genes to standard database identifiers), gene ranking based on the overall importance of each gene mentioned in the article, and gene-oriented document retrieval (identifying full text papers relevant to a selected gene). Six systems participated and all processed and displayed the same set of articles. The articles were selected based on content known to be problematic for curation, such as ambiguity of gene names, coverage of multiple genes and species, or introduction of a new gene name. Members of the UAG curated three articles for training and assessment purposes, and each member was assigned a system to review. A questionnaire related to the interface usability and task performance (as measured by precision and recall) was answered after systems were used to curate articles. Although the limited number of articles analyzed and users involved in the IAT experiment precluded rigorous quantitative analysis of the results, a qualitative analysis provided valuable insight into some of the problems encountered by users when using the systems. The overall assessment indicates that the system usability features appealed to most users, but the system performance was suboptimal (mainly due to low accuracy in gene normalization). Some of the issues included failure of species identification and gene name ambiguity in the gene normalization task leading to an extensive list of gene identifiers to review, which, in some cases, did not contain the relevant genes. The document retrieval suffered from the same shortfalls. The UAG favored achieving high performance (measured by precision and recall), but strongly recommended the addition of features that facilitate the identification of correct gene and its identifier, such as contextual information to assist in disambiguation.

**Discussion:**

The IAT was an informative exercise that advanced the dialog between curators and developers and increased the appreciation of challenges faced by each group. A major conclusion was that the intended users should be actively involved in every phase of software development, and this will be strongly encouraged in future tasks. The IAT Task provides the first steps toward the definition of metrics and functional requirements that are necessary for designing a formal evaluation of interactive curation systems in the BioCreative IV challenge.

## Background

The biological literature represents the repository of biological knowledge. The ever increasing scientific literature now available electronically and the exponential growth of large-scale molecular data have prompted active research in biological text mining and information extraction to facilitate literature-based curation of molecular databases and biomedical ontologies [1][2]. To date, many text mining tools and resources have been developed to aid in this process, and community efforts, such as BioCreative, have evaluated text mining systems applied to the biological domain [3-5]. However, these tools are still not being fully utilized by the broad biological user communities [6]. Such a gap is partly due to the intrinsic complexity of biological text, the heterogeneity and complexity of the biocuration task, and to the lack of standards and close interactions between the text mining and the user communities that include biological researchers and database curators. Previous BioCreative challenges have involved experienced curators from specialized databases (like protein-protein interaction databases in BioCreative II, and II.5) to generate gold standard data for training and testing of the systems. However, there was little focus on development of interactive interfaces for curators, and limited interaction between curators and text mining developers related to tool development. Earlier challenges involved many text mining teams in developing basic capabilities relevant to biological curation, but they did not address the issues of system usage, insertion into the workflow and adoption by curators or biologists in general. As Cohen and Hersh point out, the major challenge of biomedical text mining is to make the systems useful to biomedical researchers. This will require enhanced access to full text, better understanding of the feature space of biomedical literature, better methods for measuring the utility of systems to users, and continued interaction with the biomedical research community to ensure that their needs are addressed [7]. This was the main motivation for introducing the InterActive Task (IAT) in BioCreative III (BC-III). The long term goal of the IAT is to encourage the development of systems that address real-life curation challenges by combining multiple text mining component modules to retrieve literature and extract relevant information for integration into the curation workflow. To support the aims of the IAT in BC-III, involvement of both developers (to provide prototype systems) and end users (to assess systems) was solicited. The IAT was introduced as a demonstration task with the goal of using the results from BC-III to provide the first steps towards the definition of metrics and acquisition of data that are necessary for designing a formal evaluation of the interactive systems in the next BioCreative IV challenge. In addition, it brought together the systems developers and the biocurators, to open a dialogue between these communities.

### Related work

In BC-III, the IAT task dealt with two important aspects simultaneously: performance of the system (how accurate the results of the given task are) and usability of the interface (how user-friendly the interface is). Addressing performance of a task is the core of all BioCreative challenges. However, addressing usability is a novel aspect. Usability is important because it enables the users to find, interact with, share, compare and manipulate important information more effectively and efficiently [8]. A study on usability of bioinformatics resources by Bolchini et al. [8], has shown that usability issues were undermining the ability of users to find the information they needed in their daily research activities; issues included not understanding the result of a given search, and not understanding the ranking criteria and the content of the documents. Another usability study focused on users querying a protein-protein interaction tool and selecting items of interest from search results for further analysis. This study showed that users had certain predefined criteria to guide their judgment, and that tool designs must accord in content, arrangement, and interactivity with the user’s criteria and with way of exploring the search space [9]. There are some previous studies on evaluating the extent to which the speed of curation can be improved with assistance from text mining. Only a few systems reported greater efficiency after incorporating text mining tools within the curation workflow [10][11], whereas other studies have shown otherwise, because integrating text mining services is usually more costly than expected since wrappers and user interfaces need significant, often user-specific, development [12]. Nonetheless, all studies highlight the importance of understanding the biocurator’s curation workflow.

## Results

### Establishment of the User Advisory Group

A critical aspect of the BC-III IAT was the active involvement of the end users to guide development and evaluation of useful tools and standards. To address this, we established a User Advisory Group (UAG) by recruiting researchers actively involved in generating or using literature-based curated data, and representing diverse literature-based curation needs, especially from the biocuration field, but also including non-biocurator users (Table 1) (also see http://www.biocreative.org/about/biocreative-iii/UAG/). The roles of the UAG included i) developing the end user requirements for interactive text mining tools that were delivered to the participants in the BC-III interactive task (see task specifications below); ii) providing gene normalization annotation to a corpus of full text articles for use in developing baseline metrics (inter-annotator agreement, and time for task completion) as well as a gold standard of articles correctly annotated for gene/protein normalization (the GN task); and iii) participating in the interactive task by testing the systems, providing feedback, and attending the BC-III workshop. The UAG was consulted via monthly group teleconferences and via e-mail for further discussion of selected topics. Extra teleconferences were held at dates closer to the evaluation of the systems. Members participated at one time or another in these activities, depending upon their availability.

**Table 1:** Members of the UAG represent a diverse sample of end users with multiple text mining needs

Domains represented by UAG members and Chair*	
Model Organism Databases	dictyBase, MGI, TAIR, Gramene, Wormbase

Protein Sequence Databases	UniProtKB

Protein-Protein Interaction Databases	BioGrid, MINT

Ontologies	Gene Ontology, Protein Ontology, Plant Ontology, Microbial Phenotype Ontology

Pharmaceutical Companies	Dupont, Merck KGaA, Pfizer

**Examples of text mining needs among UAG members**	

□ gene normalization □ mapping to ontologies (e.g., GO, PO, PRO) either for annotation or semantic integration □ entity normalization and relevance scoring to help automate relationship extraction and data integration of text mined facts with external and internal sources	Identification of articles: □ related to a specific topic (PPI, biomarkers) □ reporting experimental information for gene/proteins in a given organism □ with experimental characterization of gene/protein with associated reporting of organism and gene normalization when available □ new articles not yet in the database

*Note that some members represent more than one resource

### Establishment of the IAT Task

*Defining the task*: Monthly discussions with the UAG over a period of 9 months provided the guidelines for the task described here. For the IAT evaluation, the interactivity of the task refers to the use of an interface to perform a task, with a user in the loop. In addition, the interface should provide interactive decision support, and manual selection of alternatives, with context-sensitivity to facilitate the user’s task.

This differs from “static” BioCreative evaluation tasks where systems transform input into sets of results that are evaluated against a gold standard – with no user in the loop.

The selection of the interactive task considered, among other things, the following issues:

-Shared interest in the biocuration community: Linking a gene mention to a database identifier (GN) and retrieving articles for genes with experimental information were common denominators among majority of the UAG curation activities (see Table 1). However, biocurators extract annotations for genes/proteins based on experimental data described in the literature; therefore, we introduced a ranking of genes based on relation of the gene/protein – and its species - to experimental evidence.

-Expertise of UAG members relevant to evaluate the systems: In this case the group decided to focus on a text mining task for biocuration.

-Maturity of the task: The goal was to select a text mining task with reasonable performance, such as gene normalization (GN), which has been evaluated in previous BioCreative challenges, to focus on providing the necessary features and interactive decision support to help the biocurator in the difficult curation cases.

-Time frame and team’s commitment: The task was chosen to be realistic given the time needed for developers to provide functional systems by the time of the workshop (5 months), and to encourage teams to participate and deliver in a timely fashion.

-Add some novelty to the task selected: The use of full length articles, the gene ranking, document retrieval and ranking, and request for user friendly interface with functionalities to facilitate curation were included.

Based on all these considerations, the IAT task was restricted to gene normalization (identifying which genes are being studied in an article and linking these genes to standard database identifiers) and gene-oriented document retrieval (identifying full text papers relevant to a selected gene) in full length articles (see below). Both tasks requested that systems rank results based on overall importance of the gene in the article. We believe this task still reflects a basic task shared by existing literature biocuration workflows (see Table 1 and [13]).

#### Defining the concept of centrality and gene ranking

To address the gene and document ranking criteria, the UAG discussed and defined the concept of gene centrality. The basic idea was to base the ranking on those genes associated with experimental results, as this is the feature most commonly driving literature-based annotation, and to rank these genes higher than other genes mentioned. Ultimately, the centrality concept would assist in identifying the set of genes in the article that are potentially relevant to the biocurator, and assist in ranking the genes according to overall importance in the article. In turn, this would also help in the retrieval of relevant documents about a particular gene. In the end, the biocurator would be able to know, for example, that a given article has some type of assertion about genes A, B, C, and D (although it also mentions E and F), but it is mostly about genes A and C. To come up with a consensus definition of centrality, nine members of the UAG curated the same two full length articles and selected the genes having some level of experimental information (Table 2). The exercise revealed two distinct opinions about what constituted centrality: i) genes whose experimental manipulation contributed to the main assertions of the article, versus ii) genes that were assayed in an experiment, regardless of whether they contributed to the main assertions of the article or they were markers or control proteins.

**Table 2:** Gene centrality assignment by a subset of UAG members (9) on two selected articles.

**PMC2684697**[14]			**PMC2613882**[15]		
**Gene (species)**	**Entrez ID**	**Central Vote**	**Gene (species)**	**Entrez ID**	**Central Vote**

**gata1 (human)**	**2623**	**9**	**Prp40 (yeast)**	**853857**	**9**
**gata1 (mouse)**	**14460**	**9**	**Snu71 (yeast)**	**852896**	**9**
**e2f2 (mouse)**	**242705**	**9**	**Luc7 (yeast)**	**851471**	**9**
**fog-1 (mouse)**	**22761**	**9**	ypr152c (yeast)	856275	5
**fog-1 (human)**	**161882**	**9**	DBP2 (yeast)	855611	2
**pRB (mouse)**	**19645**	**9**	ECM33 (yeast)	852370	2
pRB (human)	5925	5	Clf1 (yeast)	850808	1
CD71 (mouse)	22042	4	CA150 (human)	10915	1
	
c-kit (mouse)	16590	4			
ter119 (mouse)	104231	4			
pcna (mouse)	18538	3			
p107 (mouse)	18148	3			
beta-actin (mouse)	11461	3			
eGFP (B. cereus)	8382257	1			

The consensus genes that were considered central are in bold. Central vote is the number of UAG members who selected the given gene as central.

For example, in the case of PMC2684697 [14], gata1, e2f2, fog-1 and pRB were assigned as central genes based on their contribution to the novel assertions put forth by the authors. In contrast, genes such as CD71, c-kit, ter119, GFP, and beta-actin were mentioned multiple times in the Results section, but these were used in the experiments either as cell type markers or controls. However, the genes that were unanimously identified as central by the UAG (genes selected as central by all members, in Table 2) coincided with the view in i). In the end, the UAG agreed to define gene centrality in terms of genes whose experimental manipulation contributed to the main assertions of the article, and further agreed that an ideal system should rank higher those genes undergoing real characterization than those serving as controls or used as molecular reagents. It is important to note that in the context of this task, centrality was a binary criterion: if there were mentions of genes that were involved in some experiment (not as controls) then they were considered central. However, the amount of information content for the different genes described in the article would be different and the frequency of mention could be used to rank the genes in the context of overall importance within the article (e.g., this article is mainly about genes A and C).

#### Defining IAT System Requirements

Constraints on system requirements were deliberately kept to a minimum to encourage creativity by the participants. Nonetheless, there were fundamental functional and usability features established by the UAG:

• Populate the tool with the set of full text articles in XML format from the PubMed Central Open Access collection [16] provided by task organizers

• For the gene normalization and ranking task, the system should be able to accept as input a PubMed Central Identifier (PMCID) and display the full text with a list of gene identifiers mentioned, ranked according to overall importance in the article considering the concept of centrality (as discussed in previous section)

• For the retrieval task, the system should receive as input a gene symbol, and retrieve PubMed Central Open Access documents that mention it, ranked according to overall importance in the article considering the concept of centrality (as discussed in previous section)

• The system should provide a user-friendly web-based interface with:

✓ an editable list of gene/protein identifiers that linked out to an appropriate gene/protein-centric database (e.g. Entrez Gene [17] and Uniprot [18])

✓ a view of the full text with candidate gene mentions highlighted

• The system should also consider the following desired capabilities:

✓ support for interactive disambiguation of gene/protein mentions based on context (e.g., other genes, species, chromosomal location) to enable the user to manually select the correct unique identifier from a set of possibilities (or to enter in the identifier if it is not present in the list)

✓ ability to sort gene list based on frequency (how many times it is mentioned), location (in what sections it is mentioned), experimental evidence (whether it is studied in an experiment) or their combinations

✓ ability to collect event and timing information at the session level (and ideally at a finer granularity of user action)

✓ the ability to export results as, e.g., a tab-delimited file (a common format used post-curation to upload results to a database)

### The participating systems

*Preparation phase:* The interactive task was announced at the beginning of March 2010 and six teams registered. The teams had five months to deliver the IAT systems to the UAG for assessment (see next section). In the end, all systems provided an interface to enter a PMCID or gene name/ID to retrieve a full length article or article list, respectively, with the exception of MyMiner, which was originally designed for other purposes (see Team 61 in Methods section), but it was of particular interest to determine how suitable this system was under the BioCreative IAT task settings and to understand which features were important to the IAT users. Table 3 provides an overview of the major features of each participating system. For a more detailed description see the Methods section below.

**Table 3:** Overview of the major features offered by IAT systems.

Team	Team 61	Team 65	Team 68	Team 78	Team 89	Team 93
**System**	**Myminer**	**Odin**	**GeneView**	**U.Iowa**	**U. Wisconsin**	**GNSuite**

**Process full text**	No	Yes	Yes	Yes	Yes	Yes

**GN**	Yes	Yes	Yes	Yes	Yes	Yes

**GN task input**	Text	PMCID or PMID	PMCID or PMID	PMCID	PMCID	PMCID or PMID

**Gene ranking**	No	Yes	Yes	Yes	Yes	Yes

**Result sorting capabilities**	No	Yes	Yes	Yes	Yes	Yes

**Document retrieval based on Gene ID**	No	Yes	Yes	No	Yes	Yes

**Document retrieval based on Gene name**	No	Yes	Yes	Yes	Yes	No

**Remove or add species and/or genes (e.g., add a gene mention not detected by the system)**	Remove and add both species and genes	Remove species and genes/proteins. Add species and genes that can be associated with a term in document	No	No	Remove and add genes	Remove a gene or add it back (cannot add new)

**Link to external databases (gene mentions or species linked out to external databases)**	UniProt, NCBI taxonomy, and MIM	Entrez Gene UniProt	Entrez Gene, KEGG, UniProt, Interpro, GO, DIP, Intact, MIPS, MINT HPRD, dbSNP	Entrez Gene, NCBI taxonomy	Entrez Gene	Entrez Gene, NCBI taxonomy

**Interface data display for GN**	Multiple boxes with abstract, species and protein information	Panels with information linked interactively	Panels with information linked interactively	Panels with linked information	Table	Summary Table

**Export Results (gene list with database identifiers)**	Saves tagged abstract	Tabular format	Tabular format	Tabular format, need to specify before querying	Tabular format	Tabular format

### Assessment of IAT systems

To assess the different systems, the UAG prepared a questionnaire related to the interface usability and performance. A subset of UAG members conducted the assessment, which was done remotely. The results were collected, compared to the manually annotated set and described during the BC-III workshop. Since this was a demonstration task, not a competition, the results presented are preliminary and only a guide to evaluate feasibility of a future interactive challenge.

#### Assessing usability

**Figure 1:**
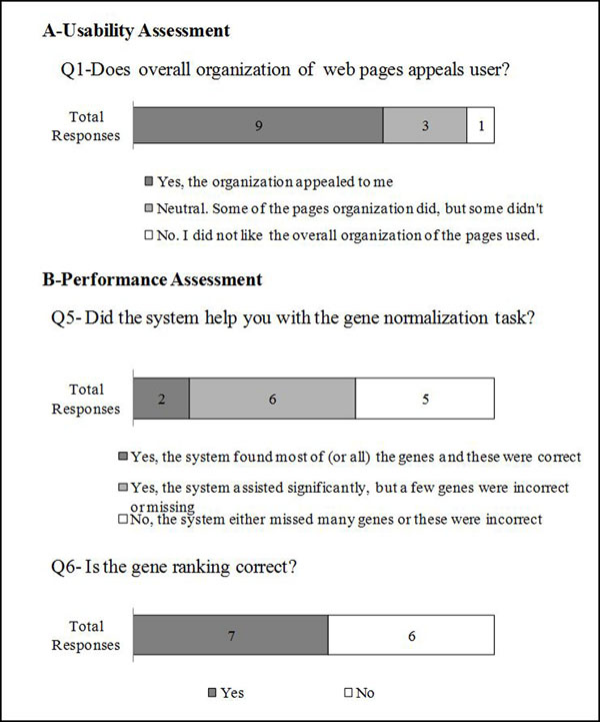
Usability and performance assessment survey results. Note that only selected questions are shown in graph format. Results are shown as number of UAG member that selected a particular response.

1. *As you operated the system interface*, *did the overall organization of the web pages appeal to you?* Figure 1A, question 1 (Q1) shows that overall organization appealed to most curators.

2. *What aspects/features about the interface appealed to you the most?* Three aspects were of common appeal to users: 1) intuitive navigation, 2) highlighting (color-coded based on entities), and 3) easy access to databases (DBs), such as UniProt, Entrez Gene and PMC.

3. *What aspects/features would you like to see added to this interface?* Two important features identified from this question were user validation (ability to add/delete species and gene names, followed by on-the-fly gene normalization and ranking), and highlighting related gene mentions and species to provide gene-species assertion evidence in the context of the full text article.

4. *List any aspects/features that did not appeal to you*. The most common unappealing aspect was species bias, which leads to inaccurate normalization, so for example in the cases analyzed, the system would link a gene mention most often to some mammalian species (usually human and mouse) even when the article did not deal with these organism at all. But even worse was the case where the systems excluded some species altogether, so it would not be possible to link the gene to its correct identifier using the given system.

#### Assessing Performance

5. *Did the system help you with the gene normalization task?* Users found that when systems correctly linked a gene mention to the corresponding database identifier, it sped up the curation process. Articles with challenging normalization examples reduced user satisfaction; Figure 1B, Q5 shows the wide-range of the responses.

6. *Is the gene ranking correct* (*i.e.*, *are the top ranked genes central*)*?* As with question 5, in some cases the gene ranking was correct, i.e., the genes with experimental characterization ranked higher than those that were mentioned in passing or were just used as markers, but the species were not assigned correctly (see Figure 1B, Q6).

The retrieval task deliberately focused on challenging gene normalization examples (e.g. *Arabidopsis* APO1 and HCF101, human WASP, and *Drosophila* TAK-1). Not surprisingly, assessment of the retrieval task, which included reviewing the top 5-10 retrieved articles for relevance to the input gene symbol, uncovered the same issues described above with correct species identification and other normalization problems. This prompted the UAG to recommend either abandoning or reassessing the retrieval task to make it independent of the normalization issues (see below for additional discussion).

### Analysis of individual articles from three use cases

To associate terms appearing in text with specific biological entities is challenging to both biocurators and systems. There are cases where different genes share the same name, even within a same species, which is a serious problem because it affects the proper identification of the gene, and, in the end, impacts its annotation [19]. It also affects the retrieval of relevant documents about the gene, with the biocurator spending time discerning what articles are for which gene. The biocurator usually looks for contextual information to assist in disambiguation, such as chromosomal location, identification of the organism bearing the gene, the mention of a synonym, and the mention of an encoded domain or its sequence length, and these same features could be used by the system to enable the user to manually select the correct unique identifier from a set of possibilities. In addition, there are multiple cases where the article introduces information for multiple genes and species, but the evidence associating genes and species is outside the sentence or paragraph containing curatable information. Sometimes Methods sections or figure legends indicate species origins via information about cDNA constructs or cell lines. In other cases the information is found in a cited reference and/or acknowledgments, but there are cases where the organism source information is simply not provided. Systems should provide whatever means necessary to help the biocurator relate gene mentions to the correct species.

Another challenging use case is the introduction of a new gene name. The curator is then tasked with capturing the new gene name, species and linking it to a database identifier. In this case it is expected that the system could link to the organism genome database if the gene is not yet annotated in multi-species gene or protein databases, such as Entrez Gene or UniProt.

With these use cases in mind, the UAG assessed the system using a set of articles that represented the selected problematic cases for curation described above, namely, gene name ambiguity, species ambiguity, or introduction of new gene names, with the main goal of assessing whether an interactive system could provide the necessary tools to assist in resolving these challenging issues. These cases are described below.

### *Case 1-* Name Ambiguity (PMC2275796 [20])

Manual and system-assisted curation of this article reveals that there are only 2 genes mentioned in the full article (inter-annotator agreement was 100% for 5 annotators using the system and 2 manual annotations), and only one of them is central (GLUT9/SLC2A9). In this case inter-annotator agreement was 100%, hence the results from curation are shown in a single column in Table 4. In this use case, the high number of false positives in systems such as systems from Team 65 or 89 is mainly due to ambiguity of acronyms shared both by gene names and clinical terminology (e.g. CAD, BMI and MI). All systems found the central gene (GLUT9/SLC2A9). However, in some of the systems SLC2A6 ranked as high as SLC2A9. Although both genes share the name GLUT9, the article clearly indicates that it is SLC2A9: “...*GLUT9* gene, also known as *SLC2A9....”* In brief, the ambiguities observed in this example could be resolved by considering contextual information. It is also worth noting that the high number of false positives may have an impact on the time consumed by the curator in curating the article. For example, the manual curation of this article by 2 curators took 15 and 27 min. Systems with low false positives (like 2-4 for Teams 78, 68 and 93) took 7 to 20 min, whereas a system with high false positives (like 15 and 42 for Team 89 and 65, respectively) took 30-48 min. Note that this is just a rough indication, and time spent on curation should be further tested.

**Table 4:** Example of an article that presents name ambiguity between gene names, and between a gene name and a term from other domain (PMC2275796).

PMC2275796			Central Vote	Curated Output^a^	System Raw Output Team				
Gene ID	Gene names	Species			78	68	65	93	89
	
56606	GLUT9/SLC2A9	human	7	Y, C	Y, C	Y, C	Y, C	Y, C	Y, C
9948	WDR1/AIP1	human		Y	Y	Y	Y	Y	-
Some examples of ambiguity found in system’s output									

11182	GLUT9/SLC2A6	human				N, C	N,C	N,C	
	CAD				N		N		
	MI				N				N
139741	MAGI2/AIP1	human			N		N		N

	Total genes detected			2	6	4	44	4	15
Performance for total of genes in the article		FP		0	4	2	42	2	14
		FN		0	0	0	0	0	1
		TP		2	2	2	2	2	1
		Precision		1	0.33	0.50	0.05	0.50	0.07
		Recall		1	1	1	1	1	0.5

	Total central genes			1	1	2	2	2	1
Performance for detecting central genes**^b^**		FP		0	0	1	1	1	0
		FN		0	0	0	0	0	0
		TP		1	1	1	1	1	1
		Precision		1	1	0.50	0.50	0.50	1
		Recall		1	1	1	1	1	1

List of Entrez Gene IDs, gene name and species found in PMC2275796. The Central Vote column indicates the number of curators that selected the gene as central; “Y”: gene mentioned in the article was detected; “-”:gene mentioned was missed; “N”: the entity detected was not a gene or a wrong gene; “C”=indicates central gene as determined by majority vote, and in the systems it means that the gene was ranked high (gene ranked higher than non central genes); “Total genes detected”: totality of gene mentions provided by a given system (what the system considered a gene). FP and FN stand for false positive and negative, respectively. ^a^Curated output by manual curation (2 curators) and system-assisted curation (5 curators) was identical so it is shown as a single column. ^b^The FP for central gene performance was calculated by comparing the list of manually curated central genes with the gene ranking by the system. If any non-central gene is ranked higher than a central one it is considered a FP.

### *Case 2-* Multiple genes and species (PMC2680910 [21])

In this case the article contains multiple genes and species, including orthologously related proteins. The inter-curator agreement in this case was lower in terms of identifying the full list of gene mentions, but the inter-curator consensus was observed for the central genes (those marked with C in Table 5). The systems identified all the human central genes, but only systems from Team 78 and 93 identified the virally encoded gag protein. In addition, systems showed improved gene mention performance (the detection of gene names is more accurate), but difficulties with species assignments contributed to increased false positives. It should be noted that although curator 5 missed a significant number of genes, s/he did not miss the most relevant ones (central). Further discussion with this curator revealed that the curator only corrected the central genes and not the entire list of genes in the article (e.g., he/she did not search for missed genes by the system).

**Table 5:** Example of an article containing multiple gene and specie mentions (PMC2680910)

PMCID2680910			Central Vote	Curated Output^a^					System Raw Output Team				
Gene ID	Gene names	Species		1	2	3	4	5	78	68	65	93	89
10015	ALIX	human	7	Y, C	Y, C	Y, C	Y, C	Y, C	Y, C	Y, C	Y, C	Y, C	Y, C
57630	POSH	human	7	Y, C	Y, C	Y, C	Y, C	Y, C	Y, C	Y, C	Y, C	Y, C	Y, C
155030	Gag	HIV-1	6	Y, C	Y, C	-	Y, C	Y, C	Y, C	-	-	Y, C	-
36990	POSH	Drosophila		Y	Y	Y	Y	Y	Y	Y	-	Y	Y
43330	ALIX	Drosophila		Y	Y	Y	Y	Y	Y	Y	-	-	Y
128866	CHMP4B	human		Y	Y	Y	Y	-	Y	-	Y	-	Y
39659	TAK-1	Drosophila		Y	Y	Y	Y	Y	-	Y	-	-	Y
3355106	ALG-2	Drosophila		Y	Y	Y	Y	Y	-	-	-	Y	-
7323	UbcH5c	human		Y	Y	Y	Y	-	-	Y	Y	-	-
1489984	p9	EIAV		Y	Y	Y	Y	-	-	-	-	-	-
137492	HCRP1	human		Y	Y	Y	Y	-	Y	-	Y	-	-
7251	TSG101	human		Y	Y	Y	Y	-	Y	-	Y	-	-
155030	p6	HIV-1		Y	-	Y	Y	-	-	-	-	-	-
7334	UBC13	human	1	Y	-	Y, C	Y	-	Y	Y	Y	-	-
	
	Total genes detected			14	19	13	26	10	90	22	120	9	52
		FP		0	5	0	0	3	81	15	113	4	46
		FN		0	2	1	0	7	5	7	7	8	8
		TP		14	12	13	14	7	9	7	7	5	6
		Precision		1.00	0.71	1.00	1.00	0.70	0.10	0.32	0.06	0.56	0.12
		Recall		1.00	0.86	0.93	1.00	0.50	0.64	0.50	0.50	0.38	0.43

List of Entrez Gene ID, gene name and species found in PMC2680910. The Central Vote column indicates the number of curators that selected the gene as central; “Y”: gene mentioned in the article is detected; “-”:gene mentioned was missed; “C”=indicates central gene as determined by majority vote, and in the systems it means that the gene was ranked high by the system (gene ranked higher than non central genes); “Total genes detected”: totality of gene mentions provided by a given system (what the system considered a gene). FP and FN stand for false positive and negative, respectively. ^a^Curated output by manual curation (2 curators, 1-2) and system-assisted curation (5 curators, but 3 are shown, 3-5).

### *Case 3-* Introduction of a new gene (PMC2764847 [22])

The last case is PMC2764847, which introduces the gene name AtHSB for the first time, along with its identifier: At5g06410: “As the name Jac1 in Arabidopsis has been assigned to another protein we named At5g06410 AtHscB”. Despite explicit mention of a database identifier in the sentence, only two systems detected this gene as shown in Table 6. In fact, most of the systems missed many of the Arabidopsis genes (see discussion). However, most of the systems successfully found the yeast central genes. There were a total of 29 gene mentions in the article (as determined independently by manual curation), but for simplicity, only the list of proposed central genes are listed (as considered by ten curators) in the example in Table 6. In this case, there were some discrepancies in the assignment of central genes with two UAG members, but these were individually discussed. In one case, the curator validated the system output, but since the system missed the *Arabidopsis* genes, these were not included (AtHscB, AtIscU1 and AtHscA1). After re-evaluating the curation, it was agreed that they should be included. Another conflict was related to two yeast genes. The problem in this case is generated by the fact that the yeast knockouts are used for complementation assays. Most curators considered these still as central because there was some information gained from the experiment about the yeast, but the article is mostly about the *Arabidopsis* genes. Note that if the systems worked as expected, the most important genes in the article would be ranked first, then the *Arabidopsis* central genes should be ranked higher that the yeast ones (this is mostly accomplished by counting the frequency of mentions in result section for these genes: AtHscB=66, AtHscA1=27, Jac1=26, AtIscU1=22, Ssq1=13).

**Table 6:** Example of an article where a new gene name is introduced (PMC2764847).

PMC2764847			Central Vote	Curated Output^a^	System Raw Output Team				
Gene ID	Gene name	Species			78	68	65	93	89
	
828316	AtIscU1	A. thaliana	9	Y, C	-	-	-	-	-
829947	AtHscA1	A. thaliana	8	Y, C	-	-	-	-	-
830529	AtHscB	A. thaliana	8	Y, C	-	Y	-	Y, C	-
852866	Jac1	Yeast	8	Y, C	Y, C	Y, C	Y, C	-	Y, C
851084	Ssq1	Yeast	8	Y, C	Y, C	Y, C	Y, C	-	Y, C
830818	HscA2	A. thaliana	1	Y	-	-	-	-	-
821316	AtIscU2	A. thaliana	1	Y	-	-	-	-	-
825719	AtIscU3	A. thaliana	1	Y	-	-	-	-	-
	Total genes detected		29 (manual)		54	22	65	9	23
		FP			46	14	58	7	16
		FN			21	21	19	27	22
		TP			8	8	10	2	7
		Precision	0.93 (0.07)^b^		0.15	0.36	0.15	0.22	0.30
		Recall	0.75 (0.16)^b^		0.28	0.28	0.34	0.07	0.24

There were a total of 29 gene mentions in the article (as determined independently by manual curation), but for simplicity, only the list of proposed central genes are listed here (as considered by 10 curators). The Central Vote column indicates the number of curators that selected the gene as central; “Y”: gene mentioned in the article is detected; “-”:gene mentioned was missed; “C”=indicates central gene as determined by majority vote, and in the systems it means that the gene was ranked high by the system (gene ranked higher than non central genes); “Total genes detected”: totality of gene mentions provided by a given system (what the system considered a gene). FP and FN stand for false positive and negative, respectively. ^a^Curated output by 10 curators (2 per system). Central genes were selected by majority vote, with previous revision of discrepancies of annotation with individual UAG members. ^b^Average value from curators output with standard deviation shown in parenthesis.

The overall assessment indicates that although the system usability features appealed to most users, there are some important features missing that are key to enhancing the system-assisted curation (see discussion section). This is relevant since the performance of the gene normalization and ranking were suboptimal, and any feature that would allow finding the correct gene and its identifier would speed curation.

A demo session during the workshop was useful for facilitating the face--to-face communication between the developers and curators, and many suggestions that came out after the assessment were promptly implemented by the systems. The results shown here, as well as the brief interaction between users and developers, indicated that the proposed task setting should be modified. In this setting the teams were given the specifications and they delivered the systems with no feedback in between, but in reality software development is an iterative process and it is critical that users and developers interact along the entire process (see discussion). This is a well-documented phenomenon in the search interface design literature [23].

### Feedback from UAG on individual systems

**Team 65:** According to the results of the IAT user experiment, the most positive characteristic of the OntoGene/ODIN system was the clear and intuitive user interface, based on dedicated panels, with information linked interactively. Negative comments regarded mostly the suboptimal organism ranking and low recall. This was partly due to the fact that the OntoGene pipeline had been originally developed for the PPI tasks of BioCreative II [27] and II.5 [28], and thus was biased towards protein-protein recognition. These limitations are currently being corrected and a public version of the system is in preparation.

**Team 68:** According to the results of the IAT user experiment, GeneView provides an intuitive and simple user interface. Providing entity specific links to external databases is also regarded as a convenient function for manual curation. The most requested feature is the possibility to manually correct (add, remove or edit) genes. Team 68 is currently working on an enhanced version of GeneView, which will include more entity types with the capability to modify annotations.

**Team 78:** According to the results of the IAT user experiment, the organization of information was appealing, especially, due to the presence of contextual coloring for genes and species and easy access to external databases. A majority of the UAG members agreed that the system would assist in the gene normalization task with the top automatically-ranked genes being the central ones. Among the desired features are the ability to validate, suggest or delete gene names for an article and higher system recall. The former feature was disallowed due to system security and integrity concerns as a malicious or novice user might make undesirable modifications to the database. Team 78 is working on improving the algorithm to achieve better recall and these changes will be gradually integrated into the system.

**Team 89**: According to the results of the IAT user experiment, the overall performance of Team 89 at IAT was mediocre. This was partly due to the performance of the gene normalization system. The interface’s speed and ability to add and delete genes was appreciated. However, the inability to view the genes highlighted in the article alongside the table of identified genes was seen as a major limitation. The default ranking of the genes based on a machine-learned centrality score often favored genes from well-studied species such as humans and mouse, and was often uninformative. A simpler approach of sorting genes by frequency would have been preferred. The comments received from the UAG are being addressed.

**Team 93**: According to the results of the IAT user experiment, the most positive characteristic of the GNSuite system was the clear and intuitive user interface with nice table layout and context information color-coded interactively. Negative comments mostly concerned the bias towards human genes and the high error rate. These problems can both be addressed by ignoring/removing the MEDIE input (responsible for most false positives), or by replacing/adding new and better GN sub-systems as they become available. The team is working on making module switching straightforward by using stand-off notation and common identifiers. The system was not stable in the beginning of the test phase, but this was fixed prior to the workshop.

**Team 61:** According to the results of the IAT user experiment, of particular interest to end-users are the flexible editing of automatically recognized bio-entities and the option to select specific species of relevance. Aspects that would improve MyMiner in future developments include recording of previous choices (prefilled choice box) of the users through the use of a user-task management system or the capacity to add user-provided customized bio-entity dictionaries.

## Discussion

The discussion is divided into three sections. In the first section, we describe common bottlenecks in the curation process culled from the literature and UAG feedback. In the second section, we suggest features that address these bottlenecks. In the third section, we suggest changes to the overall interactive task based on the experience from BC-III.

### Curation bottlenecks and potential solutions

Unassisted and assisted curation by UAG members highlighted a number of curation issues, many of which have been noted in other descriptions of curation workflows [1,2,24]. Table 7 classifies the typical curation challenges. When faced with an unrecognized gene synonym (i.e. a false negative), the impact on curation is reduced recall. Reasons for unrecognized synonyms varied. Synonyms found by some systems and not others reflected the number of gene/protein-centric databases that systems consulted for the gene normalization task. Some synonyms were not found in any database, either because authors introduced new synonyms, or a new homolog in a particular species was introduced, and the gene name was appended to a prefix to indicate species, e.g. AtHscB to indicate the *Arabidopsis thaliana* isoform of HscB (PMC2764847).

**Table 7:** Gene Entity Recognition errors and potential solutions

Error Class	Error	Example (PMCID)	Potential Solution
Synonym Not Found	New synonym is not found in databases	AtHscB (PMC2764847)	Increase breadth of databases searched by tool
	
	Species prefix obfuscates synonym	AtHscB (PMC2764847)	Ability to add synonym or species-specific rules for string matching

Ambiguity	Synonym is a common English word	WASp	Ability to add or remove a synonym and reprocess highlighting
	
	Synonym maps to more than one identifier	AIP1	Present matches simultaneously with clues like other synonyms and interacting partners
	
	Species not clearly specified	Reference 5 in PMC2680910	Be able to navigate to other sections of the paper, other papers; be able to curate to orthologous cluster of proteins
	
	Synonym refers to a protein family or an enzymatic activity		Ability to curate to protein family or orthologous cluster of proteins

Ambiguity is the other major source of curation inefficiency with potentially greater impact. Consider the case of GLUT9, a frequent synonym and primary topic of PMC2275796 (see Table 4). Given a choice between two unique identifiers (human SLC2A9 and SLC2A6) that share GLUT9 as a synonym, if the system chooses the wrong identifier, it generates a false positive result (decreased precision) as well as a false negative result (decreased recall) for the correct identifier that was overlooked. Causes of ambiguity are well-studied and have been described elsewhere [19,25,26], and it was a common phenomenon in the papers used for the IAT. One of the findings by the UAG was that the cause of ambiguity influenced how best to resolve it, which is covered in the “Recommendations to Interactive Systems Developers” section below. Lack of species specification is a notable source of ambiguity [1]. During the curation of papers used for the IAT, it was noted that a protein mention lacking species in an article introduction referred to references for more than one species (e.g. in PMC2680910, reference 5 reviews eukaryotic components of the vesicle-trafficking network). We hypothesize that named entity recognition of proteins can be deliberately vague for several reasons: to suggest that an experimental finding applies across species, or to make concise the description of a complex experiment using proteins whose origins are described in another section of the article.

### Recommendations to interactive system developers

The demonstration interactive task provided curators from different databases with varying levels of experience the unique opportunity to view the same full text articles in systems with different features. This made it possible to identify individual features that contributed to or detracted from the gene normalization task. The recommendations below are based on user feedback. The aim of this section is not to prescribe specific features, a few of which are included to clarify recommendations. Rather, the recommendations are intended to outline a general need that can be implemented any number of ways in an interactive system.

*Juxtapose contextual clues with as many candidate solutions as possible to simplify decision making.* When faced with a proposed gene mention, the curator must use contextual clues to decide which identifier to assign. These clues include other terms in the sentence in which the mention is found and references cited by the sentence. Consider the following article title: “AIP1 mediates TNF-alpha-induced ASK1 activation by facilitating dissociation of ASK1 from its inhibitor 14-3-3” (PMC161425). At the time of this writing, AIP1 alone is a synonym for eight human genes. If a curator is forced to open a separate browser window to investigate each of the eight alternatives, he or she must recall the context around AIP1. Systems like Reflect [27] offer a promising alternative. Hovering the cursor over the candidate synonym causes a pop-up window to appear where the user can cycle through all eight options and view synonymous terms, chromosomal locations, subcellular localization and other information. One of the eight genes has the synonym, “ASK1-interacting protein 1”, an excellent candidate given the contextual clues for ASK1 in the title.

The simplest way to resolve ambiguity differs from case to case. A system that presents a comprehensive view of a gene or protein, including synonyms, definitions, chromosomal locations, or interacting partners, has a higher probability of providing the clue that pinpoints the correct gene identifier. Using the GLUT9 example from PMC2275796 mentioned previously, the article is about GLUT9 polymorphisms and their association with symptoms of gout. The adjacent gene WDR1 is mentioned, so a system that presents chromosomal locations of candidate genes will display 4p16 for both, providing the curator with solid evidence for assigning an identifier. Ideally, systems can capture curatorial decisions to retrain gene normalization algorithms. Curators will accept or rejects gene calls outright, they will select from a set of suggested identifiers, or they will exit the system to find the correct identifier. Each of these actions provides critical feedback with respect to algorithm performance and coverage of external sources of identifiers.

#### Within an article, group mentions of the same gene with context for each mention and propagate curation decisions for a synonym across the article

Although gene and protein names are notoriously ambiguous, there is typically a single meaning in a document. By viewing all the text excerpts that mention an ambiguous term from one paper, the user has more contextual opportunities to resolve the ambiguity. For instance, the ninth mention of GLUT9 in PMC2275796 has the context, “the *GLUT9* gene, also known as *SLC2A9*”, thereby resolving ambiguity for all previous and subsequent mentions in the article. Similarly, if a synonym is erroneously assigned to the wrong identifier, it will result in numerous errors that can be corrected by a single fix. Therefore, curation systems need to be able to accept revisions on a per term basis and propagate them throughout the document.

#### Query as many sources as possible using as many kinds of identifiers as possible

Some incorrect gene calls, whether they were missed outright or were attributed to the wrong species, were very obvious to curators due to unambiguous identifiers or explicit species mentions in the title of the article or in adjacent sentences. One of the test articles (PMC2764847) contained an unambiguous identifier adjacent to the introduction of a new gene symbol (“we named At5g06410 AtHscB”), but none of the systems detected At5g06410 as a unique identifier from TAIR [28], the only database that contained the identifier at the time of the BioCreative workshop. This suggests that participating systems left out some sources of gene identifiers. The same article explicitly states “Arabidopsis” in the title. Coupled with the nomenclature convention of preceding homologues with the initials of the genus and species (e.g. “At” for *Arabadopsis thaliana*), a simple heuristic should eliminate some false negatives.

#### Allow for non-species-specific gene mentions when the author generalizes across species

The molecular target of thalidomide, a severely teratogenic therapeutic compound, was recently discovered to be the cereblon protein using biochemical approaches [29]. To demonstrate the role of cereblon in development, the authors used zebrafish, chick and mouse systems to assemble compelling evidence for how thalidomide administration to pregnant women could have caused the severe limb deformities witnessed in the 1960’s, an experiment that is otherwise unethical in human systems. The authors’ concluding sentence in the abstract (“Thalidomide initiates its teratogenic effects by binding to CRBN and inhibiting the associated ubiquitin ligase activity”) deliberately excludes species references to generalize their findings in lieu of a definitive experiment. A curation system that can aid the capture of these findings might look to the Protein Ontology [30] or the Clusters of Orthologous Groups (COG) database [31] as an alternative to species-non specific database identifiers.

#### Show a record of changes and allow for reversing decisions

If a curator works through a set of proposed gene mentions during article curation, the ability to tell which suggestions were accepted outright, which ones were changed, and which ones have not yet been evaluated relieves the curator from recalling each decision, especially if curation takes place over a matter of hours or days. This suggestion is the direct result of a feature from the GNSuite system (Team 93).

### Recommendations for the Interactive Task challenge

The demonstration task and ensuing discussion not only highlighted some of the curation challenges; they also helped to crystallize how an interactive task can be run as a challenge in BioCreative IV. The aim of this section is two-fold: to make specific recommendations for how the challenge should be run, and to identify critical topics overlooked in the demonstration task and gather the necessary expertise to refine the IAT design.

#### Pair developers with curators throughout the process

The workshop session where developers showcased their systems to curators elicited feedback that could have been rapidly integrated into the systems to improve their performance. Since the software engineers working on these tools generally do not have biological knowledge, it can be difficult for them to know features in which to invest effort. Clearly, some guidance based on curation expertise earlier in the process should lead to better results.

#### Encourage systems to adopt an interoperability standard to allow direct comparison of gene normalization algorithms

Performance and usability are distinct yet equally important aspects of the interactive task. In the demonstration task, it was difficult to separate the two. The systems differed in their proposed gene identifiers, which distracted curators from commenting on the curation features themselves. If systems were sufficiently interoperable such that they could make use of any number of gene normalization modules, it would be trivial to eliminate user bias based on differences in gene normalization performance, allowing curators to focus on usability.

#### Reassess the document retrieval task

The demonstration task required that systems provide the ability to enter a gene synonym and retrieve papers that mention it ranked by centrality. We propose reassessing how this feature is incorporated for several reasons. First, although this functionality as originally conceived was intended to retrieve relevant articles for a given gene that may be of significance for the curator, it may not fit in the real curation workflow. Many databases have their own triage process to retrieve the articles to curate, and this process may be uncoupled from the curator's activity (i.e., the curator works on the set of articles that have been already selected).

Second, centrality proved to be challenging to define for the retrieval task, making it difficult to evaluate systems’ retrieval performance consistently. Lastly, information retrieval and document ranking involve different algorithms than gene normalization. We suggest further discussions with a broad base of biocurators about realistic applications of a document retrieval task and how they fit with typical curation workflows.

#### Set evaluation metrics

User interface evaluation is a field of study unto itself [23] and UAG members had no formal expertise in this area. In order to transform the Interactive Task from a demonstration task to a challenge task, we recommend bringing in usability evaluation experts to more effectively communicate the specification expectations and judgement criteria prior to the challenge. For instance, we did not explore recording software to capture mouse clicks and navigation within and outside systems. Presumably, a self-contained system that aids ambiguity resolution without having to navigate to other sites will result in speedier curation. We would like to explore how tracking software could be converted into quantitative data by which system performance can be measured and compared.

Finally, we have not discussed novelty as an exploitable curation feature. Clearly, a system that can compare findings from incoming documents to existing curation and prioritize the documents that have new findings will be of great utility. During UAG discussions, database representatives voiced the need for a system that could compare the content of an article in the curation queue to existing database content and highlight articles that contained missing information. Determining the feasibility of incorporating this into an interactive challenge will require more discussion among developers and system administrators of curated literature databases.

In sum, the IAT was an informative exercise that advanced the dialog between curators and developers and increased the appreciation of challenges faced by each group. The recommendations that emerged will help to focus and inspire future developments, and they will encourage debate and discussion between distinct disciplines. The resulting systems have the potential to address major issues with biocuration: they could significantly aid in addressing the backlog of uncurated articles that should be added to existing literature-based databases; systems might emerge to help authors create structured digital abstracts [32,33]; and biocuration from novices might be improved by refining some basic tasks such as gene normalization.

## Methods

The full text articles in XML format from the PubMed Central Open Access collection was made available to participant systems at http://www.biocreative.org/resources/corpora/biocreative-iii-corpus/

### System assessment method

A total of ten UAG members (including the chair) participated in the system assessment. The systems were tested against the same set of articles (five articles in total). One of these articles was common to all members and used for training so they could familiarize themselves with their assigned system. For this, an article previously curated by all group members was selected (PMC2613882, the subject of Table 2). Each of the systems was primarily assessed by two members, with each member curating a different set of two articles which were novel to them. The exception to the assessment procedure above was MyMiner which was inspected separately as it was not originally designed to meet the specifications of the IAT task. The assessment of all systems was done remotely. The UAG members curated the articles using the system: they would get the raw output from the system, go over the gene list provided by the system and add any missing genes, correct mis-assigned organisms, and identify central genes. Once the initial assisted-curation task was complete, curators were permitted to use and comment on other systems. Note that there were some limitations to testing, including assignment of two curators per system and the number of articles processed, due to time constraints (only 2 weeks), and number of UAG members that participated in the testing (not all were available). UAG members recorded the time spent curating using the assigned system. The latter activity could not be reliably compared in all cases because some of the UAG members timed their annotation for validating central genes, while others timed their activity for validating all genes. However, in one case we can provide some preliminary information based on comparison to the manual, unassisted time spent for curation (see case 1 in Result section).

For performance assessment the precision and recall for the gene normalization task were calculated as follows:

Precision = TP/(TP+FP)

Recall= TP/(TP+FN)

Where,

TP: true positives, i.e. number of genes correctly identified and linked to the correct database object.

FP: false positives, i.e. number of gene mentions that are incorrectly identified, including cases of gene mentions with incorrect database link (mis-assignment of species), and non-gene mentions (mentions that are not genes but are detected as such by the systems and/or curators).

FN: false negative, i.e., number of missed genes (not detected by systems and/or curators).

Further information about the IAT task is available at http://www.biocreative.org/tasks/biocreative-iii/iat/.

### Systems description

#### Team 65- ODIN (Simon Clematide and Fabio Rinaldi)

**Figure 2:**
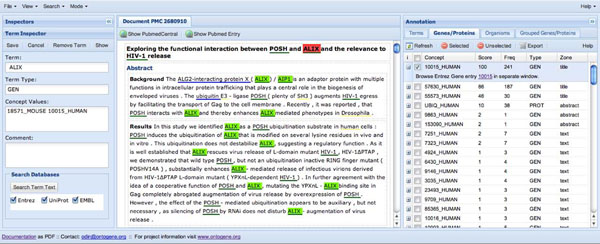
ODIN interface. The ODIN interface is organized in 3 panels: the inspector panel (left) is used to edit single annotations, the document panel (center) contains the document being inspected, and the annotation panel(right) contains grid views (in different tabs) of the terms, concepts and interactions identified by the system in the target document. The term tab contains columns showing the textual form of a term occurrence, its possible concept identifiers and main semantic types together with an ambiguity count. In the concept tab (called "Genes/Proteins" for this task) there is a row for each concept identifier with a relevance score, a frequency count, the most prominent text zone where the concept appears (title, abstract, text), its semantic type, and a link to allow exploration of the concept in the web interface of the ontology where it stems from.

URL: http://www.ontogene.org/odin/ (Figure 2)

The ODIN system is being developed within the scope of the OntoGene project, as acollaboration between the OntoGene group at the University of Zurich and the NITAS/TMS group (Text Mining Services) of Novartis Pharma AG. The purpose of the system is to allow a human annotator/curator to leverage the results of a text mining system in order to enhance the speed and effectiveness of the annotation process.

*Methods:* The OntoGene system takes as input a document in plain text or supported XML-based formats (including PubMed Central) and processes it with a custom NLP pipeline, which includes Named Entity recognition and relation extraction. Entities which are currently supported include proteins, genes, experimental methods, cell lines, and species. Entities detected in the input document are disambiguated with respect to a reference database (UniProt [18], Entrez Gene [17], NCBI taxonomy [34], PSI-MI ontology ). Since ODIN was primarily intended as a document inspector for annotation purposes, there is only an experimentally added retrieval function without ranking of the results.

Interface: The annotated documents are handed back to the ODIN interface (as pure XML documents), which allows multiple display modalities, plus various selection and modification options. The curator can view the whole document with in-line annotations highlighted, or can browse the extracted entities and be pointed back to the mentions within the document. All entity annotations are editable. Different entity views are supported, with sorting capabilities according to different criteria (entity type, confidence score, etc.) Selective display of text units (e.g. sentences) containing entities of interest is supported. Rapid disambiguation can be achieved through manual organism selection. Additionally, extensive logging functionalities are provided, which may be integrated in the document itself for document revision purposes. More details on ODIN are available in additional file 1.

#### Team 68- GeneView (Philippe E. Thomas and Ulf Leser)

**Figure 3:**
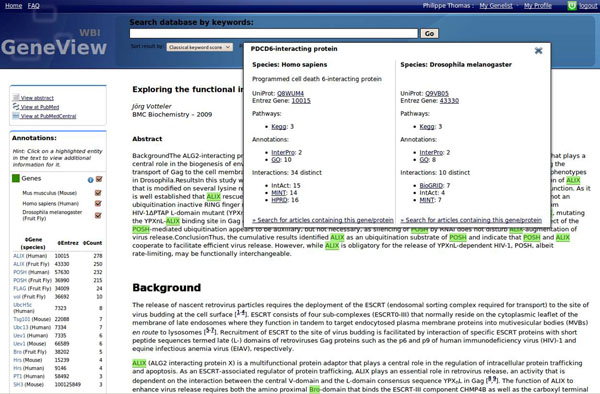
GeneView interface. The main panel shows the article and the recognized entities. Detected gene names are highlighted in green and entity-specific information, as shown for gene ALIX (PDCD6IP), is displayed. The left panel provides an overview of all entities found in the article sorted by overall count. This ranking can be manually modified. Per default all genes are highlighted in the text, but GeneView allows to limit the highlighting to the species of interest.

URL: http://bc3.informatik.hu-berlin.de/ (Figure 3)

GeneView is a tool for gene-centric searching, ranking, and visualization of scientific full text articles.

*Methods*: GeneView initially performs a series of pre-processing steps on each corpus that should be indexed: Full text articles are parsed and indexed using Lucene. Gene names are identified and normalized to Entrez Gene IDs using the BioCreative III version of GNAT [35,36]. This version of GNAT has been improved to deal more efficiently with full texts and allows for a more general species-specific disambiguation of gene names. In addition, single nucleotide polymorphisms are identified using MutationFinder [37]. All recognized entities are added to the Lucene index, together with the section type they were found in and their entity type. This structure allows for a very fast, section-specific search for entities, words, or phrases, and is also used for section specific article ranking.

To find articles that are most relevant for a given gene, the gene index and the sections in which the gene appears are taken into account, as suggested in [38]. Approximately 2,000 different section boost settings using the NCBI Gene2Pubmed mapping as gold-standard have been evaluated. Precision of each setting has been estimated using 10 randomly selected genes and their top 20 query results. On this subset the team achieved an overall precision of 72.2%. Using the best section-specific boosting, precision increased by 3.5%. This setting reflects our assumption that sections like Title, Abstract and Result are of higher importance than other sections. Surprisingly the incorporation of figure and table captions decreased the quality of ranking.

*Interface:* HTML-based display of an article encompasses the full text itself with highlighting of all identified entities and a count-based summary of detected entities. Users can access entity-specific information, integrated from a number of public data sources, by a single mouse click. As the importance of genes mentioned in the article depends on a specific user's needs, GeneView allows personalization of the ranking function. Per default, genes are ranked by their total number of occurrence in the article, but users have the possibility to exclude sections from this calculation.

The processing time for a query is currently less than one second. To further assist user in assessing the relevance of an article and its contained genes, GeneView also identifies all genes co-occurring with a given query in any of the articles in the corpus. Each such gene is tested for positive association using a single sided χ^2^-test. The five most significantly associated entities are then displayed by GeneView at the top of the search results page.

#### Team 78- University of Iowa (Sanmitra Bhattacharya and Padmini Srinivasan)

**Figure 4:**
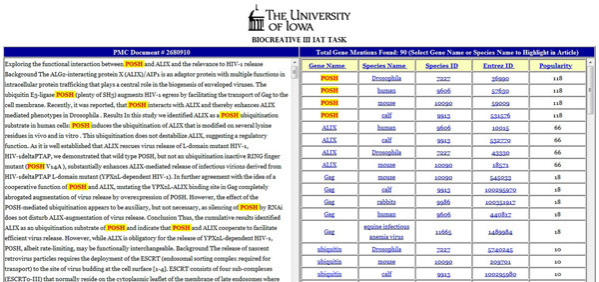
IAT interface from University of Iowa. The left panel displays the full text of the article selected by the user for the purpose of gene normalization. The right panel shows a ranked list of gene and species names along with their normalized identifiers. In this figure, all instances of the user-selected gene *POSH* are shown to be highlighted.

URL: http://siena.cs.uiowa.edu/~biocreative/ (Figure 4)

The system for the IAT task [39] was developed based on the corresponding BioCreative III gene normalization system [40].

*Methods:* The gene and protein mentions were identified in the full text using ABNER [41] and LingPipe [42] while the species mentions were identified using LINNAEUS [43]. The initial gene list was filtered using a stop list of terms (e.g. ‘antigen’, ‘*Ab*’, etc.) and shorthand gene names were expanded to constituent terms. Also the LINNAEUS species dictionary was modified to include genera of model organisms (e.g. *Arabidopsis* for *Arabidopsis thaliana*, ID: 3702) and common species strains (e.g. *Saccharomyces cerevisiae S288c*, ID: 559292). Gene and species entities were then associated if they appeared within fixed character windows and the resulting pairs were searched on the Entrez Gene database. The first Entrez Gene hit obtained from a search is returned as the unique identifier for a particular gene mention.

*User Interface***-** The interface of the system for the IAT task is simple and intuitive. Users have a choice of selecting inputs for either the indexing or the retrieval subtask. For the indexing subtask, the full text of a user-selected article is displayed in the left frame of the web page. In the right frame the gene names, species names, normalized NCBI Taxonomy IDs, normalized Entrez Gene IDs and frequency count of the gene names corresponding to the article are displayed. The results are pre-sorted by the frequency count which is based on the count of the gene names as identified by the gene name taggers. However, users may sort the results on individual fields. The gene and species names are highlighted in the full text in yellow on selecting the individual gene and species names from the right frame. The species identifiers and normalized Entrez Gene IDs have linkouts to corresponding records in the NCBI Taxonomy database and the Entrez Gene database, respectively. For the retrieval part of the task, the system displays a sortable list of PMCIDs with the frequency of the selected gene mention for that article. Each PMCID of the list has link to the full text of the article.

#### Team 89- University of Wisconsin (Shashank Agarwal and Feifan Liu)

**Figure 5:**
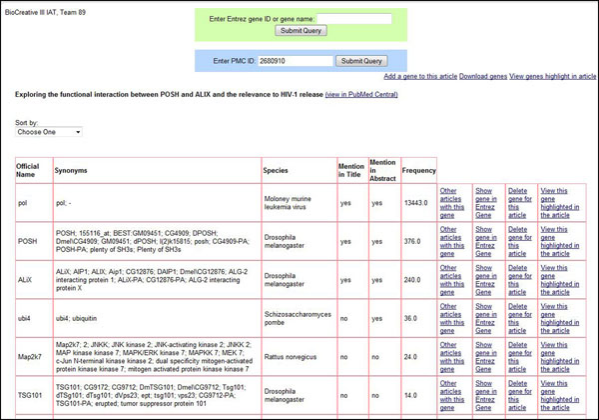
GeneIR interface from University of Wisconsin. Screenshot showing the two search boxes. Results are presented as a table. Links are provided to view the genes highlighted in the article, add or delete a gene and download the gene list. List of genes can be sorted by centrality (default), presence in title and abstract, or the frequency with which they appear in the article.

URL: http://autumn.ims.uwm.edu:8080/biocreative3iat/ (Figure 5)

Team 89 developed a demonstration system-GeneIR, that performs both gene indexing and gene oriented document retrieval.

*Methods*: For gene normalization, a machine learning system was developed. The system used existing named entity recognition tool (Banner) to identify gene mentions and employed information retrieval based method to map those mentions to their candidate genes in Entrez Gene database. To further disambiguate the candidate genes, several learning algorithms were explored. A variety of features, such as the gene’s species’ mention in the article, presence of a part or whole of the gene’s genetic sequence in the article, and similarity between the gene’s GO [44] and GeneRIF [17] annotations and the article, were used for model training.

For article retrieval, all articles in the data source were indexed by different fields such as article’s title, abstract, full text, figure legend and references, which offerflexible support on different retrieval strategies as well as interface functions. To account for gene name variations (for example, BRCA1 vs BRCA-1), a gene name variation generator was implemented. For a gene name query, the system matches it and its variations to the index for article retrieval. For a gene ID query, the system obtains the gene's symbol and synonyms and uses them along with their variations as query to retrieve relevant documents.

*Interface*: A user interface that provided two search boxes was developed: one to obtain articles based on gene name or gene's Entrez Gene ID, the other to obtain all the normalized genes from an article of a given PMC ID. From the gene results or article results, one could view other genes in an article or other articles containing a specific gene, respectively. When viewing the gene normalizations from an article, the genes can be sorted by centrality (default), presence in title and abstract, or the frequency with which they appear in the article. To determine the centrality of a gene, a machine learning classifier was trained that makes use of features such as the presence of the gene’s mention in title or abstract, the frequency of the gene’s mention in the article, and the popularity of the gene in public resources GO and GeneRIF. The interface allows users to be able to view all genes or an individual gene highlighted in the article, as well as manually adding or deleting genes from a given article. The displayed gene list can be downloaded as a tsv (tab separated values) file.

#### Team 93 - The GNSuite system (Rune Sætre and Naoaki Okazaki)

**Figure 6:**
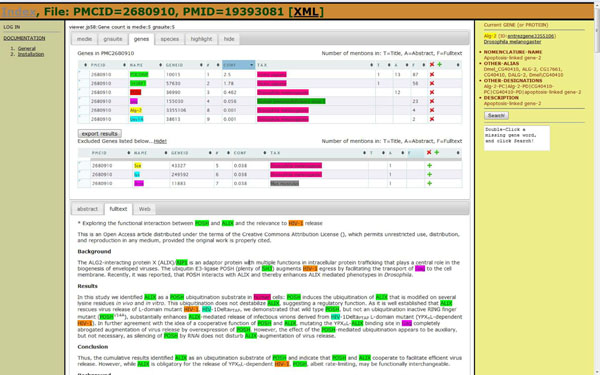
GNSuite interface. A screenshot for PMC 2680910 with the “gene summary table” and “full text” tabs activated. On the left are links to the system documentation, and on the right is detailed information about the most recently clicked gene name. On the top of the screen, right under the PMC and PubMed identifier information, are tabs for the different input sub-systems for genes and species information in addition to the summary tabs and a “hide gene tables” tab. The gene table can be saved locally by clicking the provided button. On the bottom of the screen are three tabs for viewing the abstract/MEDIE or full text/GNSuite or Web-search results respectively. The selected gene and species names from the top tables are highlighted in the texts at the bottom.

URL: http://www.idi.ntnu.no/~satre/biocreative/IAT

http://www-tsujii.is.s.u-tokyo.ac.jp/satre/biocreative/IAT/ (Figure 6)

*Methods:* The GNSuite service is running on two servers in different parts of the world for efficiency and stability. The GNSuite web-based interface is used to present pre-processed input from the underlying parsing, protein recognition and DB identifier assignment systems. Eighteen thousand full text articles are indexed by GNSuite, and more than eighteen million abstracts from PubMed by MEDIE [45].

The system accepts several sources of input such as, MEDIE , GNSuite, and LINNAEUS. This can easily be extended with other systems that provide stand-off annotations, since each system is presented in a separate tab in the user interface. All underlying results are integrated to improve recall. A web-service [46] is used to find and highlight alternative names for the recognized genes and species in the text. See the BioCreative III Gene Normalization article for more details on the GNSuite sub-system (Look for Team 93 in the GN article in this BC-III issue).

*Interface:* The GNSuite front page shows PMC and PubMed identifiers for all the available full text articles (sorted, and grouped into several pages). The number of normalized genes found in the title/abstract/full text for each article is also shown.

A “gene table” tab summarizes and ranks the recognized genes based on the combined input from all the underlying systems. This list of genes for all articles can be sorted by relevance scores based on frequency, confidence, whether they appear in the title or abstract, etc. On the top of each article’s individual visualization page (Figure 6) is a summary table with all the genes and the number of mentions in the article. The user can click on any gene symbol to see the entry in Entrez Gene, and all the recognized gene names are highlighted in the text. The user can jump from one gene occurrence to the next by clicking on the gene name, either in the abstract or in the full text. The gene table can be manipulated both manually and automatically, and can be stored to a local file on the user’s computer.

#### Team 61- MyMiner (David Salgado and Martin Krallinger)

**Figure 7:**
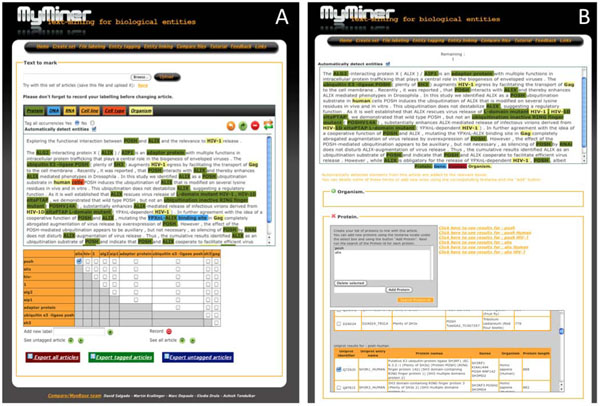
MyMiner interface. MyMiner Entity tagging and Entity linking user interfaces for PMC2680910 article abstract. Entity tagging (A) and Entity linking (B) have been manually edited; some tags have been added or removed depending on the bio curator choices.

URL: http://myminer.armi.monash.edu.au (Figure 7)

The MyMiner project proposes a set of tools (1) that facilitate individual and community-based annotation initiatives, through a free and user-friendly interface that performs the most common tasks in manual literature curation and dataset creation; (2) that aim to improve performance of predictive systems, by enhancing the quality of manually annotated sets of documents required for the development of text-mining applications; and (3) that simplify the transfer of unexploited knowledge encoded into textual format within scientific documents into computer-usable information. MyMiner has been instrumental for the creation of a muscle-dedicated database and during the BioCreative III PPI project to classify scientific documents, gene ontology terms and disease descriptions, to detect and normalise bio-entities (e.g. genes and proteins) embedded in text and to detect protein-protein interactions.

*Methods:* The MyMiner system works with any input text and thus was not tailored to specific format of the set of articles proposed by the task organizers. It is based on a general 3 column tabulated input format that allows MyMiner to be utilized by users with limited computer skills. The recognition of bio-entities is based on the integration of the named-entity recognition tool ABNER, that automatically tags mentions of proteins, genes, cell lines, cell types (ABNER). LINNAEUS is used to recognize the species. In order to generate from an entity tagged text a ranked collection of database links, MyMiner proposes a list of database identifiers per bio-entity mention. We use the UniProt query scoring mechanism for proteins and genes [47]. In this case, the protein mentions that are either automatically or manually tagged are used as direct queries within MyMiner to retrieve a ranked set of hits. Alternatively, organism query filters can be applied. The main features that influence the scoring/ranking mechanism are: (1) How often the term (i.e. selected gene/protein mention) occurs in a given UniProt entry (not normalizing with respect to the document size to avoid over-weighting sparsely annotated records), (2) Weighting depending on the field of the record in which the term was detected (e.g. higher weights are returned for hits against the protein name fields as opposed to a referenced publication field); (3) Weighting depending on whether the record had been reviewed or not, scoring higher those records that have been reviewed (as they are generally more reliable); (4) Weighting depending on how comprehensively annotated a record is, to deliberately bias the system for well-annotated entries, which in general are also more likely to be the actual hit given an input article. Ajax requests are executed to query distant databases such as NCBI taxonomy, Uniprot and OMIM [48] databases, using web services protocols or similar. Results of theses queries are treated and displayed “on the fly”, on the webpage.

*Interface:* The MyMiner application combines several standard web languages and techniques such as PHP, Javascript and Ajax to enhance user interactivity. MyMiner is composed of four main application interfaces: “File labelling”, “Entity tagging”, “Entity linking”, and “Compare file”. MyMiner user interfaces offer options and tools to resolve a variety of limitations and bottlenecks identified in each task. To make this system flexible and interactive, automatically generated tags can be corrected, edited or removed. Entities are highlighted using CSS and Javascript. When a tag is defined, a corresponding CSS style is dynamically created. Upon user actions, such as text selection and tagging, html tags are added using Document Object Model manipulation functions in Javascript. Each module provides an export option to save results. The time spent for processing a document is recorded and available on the export file. To enhance the user-friendliness of interfaces, a common display layout has been adopted and conserved between applications. Text area that contains the text or document to be analysed is located on the top of the page. Options and tools are placed below the main curation zone.

***MyMiner applications relevant to IAT task-*** The module, “Entity tagging” allows the automatic tagging of entities of biological interest in a document. It enables the manual correction and editing of those terms to overcome potential tagging errors and facilitates user interaction. Moreover, the user can add new terms, and specific relations between terms using a matrix check box. Such relations might be useful for the extraction of annotations, e.g. protein-protein interactions or protein functions.

The “Entity Linking” module facilitates the identification of database links for proteins, species and diseases mentioned in a document. Biological terms are first automatically detected and displayed in a list that can be manually edited to add new terms or to remove incorrectly identified ones. MyMiner then links each identified gene/protein to UniProtKB identifiers. A check box allows the selection of the most appropriate identifiers from the list of potential candidates. A short description is provided for each term to help validate those candidates. Species and diseases are mapped to NCBI taxonomy and OMIM database identifiers, respectively. Help sections and tutorial movies are provided. A feedback form is also available to send comments and suggestions.

## Competing interests

The authors declare that they have no competing interests.

## Authors' contributions

CNA and PMR drafted all sections in the article except the system descriptions. CNA, PMR, GC, AC, PG, MGG, IH, EH, DL, ZL, LM, LP, LT participated in the design of the IAT task, the systems assessment, article curation, and interpretation of results. SA, SB, SC, MK, UL, FL, NO, FR, PS, RS, DS and PET provided the IAT systems and wrote the corresponding system descriptions. CNA, CHW and LH were the organizers of the IAT task and oversaw the whole process. All authors read, edited and approved the final version of the manuscript.

## Supplementary Material

Additional file 1 More details on system descriptions. Click here for file
