# Supplementary material for: BioCreative III interactive task: an overview
Source: BMC Bioinformatics. 2011 Oct 3;12(Suppl 8):S4. doi: 10.1186/1471-2105-12-S8-S4 (PMC3269939; doi:10.1186/1471-2105-12-S8-S4)
Supplement: Additional file 1 — More details on system descriptions. [file 1471-2105-12-S8-S4-S1.docx]

**Supplemental Material**

**System specifications**

**Team 65-Odin**

The curation interface is mainly developed as a JavaScript-based web application using the extjs JavaScript framework, which supports rapid prototyping. Visualization is very flexible and user-adaptable through CSS and DOM manipulation. Table or grid views present attributes of interesting entities (terms, concepts, organisms, etc.) in a spreadsheet-like manner: reviewing, sorting, filtering, and removal of entries can be performed very effectively. The user can choose which level of analysis should be presented to him, e.g. textual form of terms or concept identifiers. For term tables, Odin has columns containing the textual form of a term occurrence, its possible concept identifiers and main semantic types together with an ambiguity count. For concept tables (called "Genes/Proteins" for the task at hand), for each concept identifier there is a relevance score, a frequency count, the most prominent text zone where the concept appears (title, abstract, text), its semantic type, and a link to explore the concept in the web interface of the ontology where it stems from. Much care has been invested to enable unobtrusive, but flexible term spotting and highlighting, as well as intelligent linking of information. The fact that ODIN is a web application allows extensive combination of document-internal information with knowledge retrieved dynamically from external locations, such as reference databases.

**Team93-GNSuite**GNSuite full-text results are integrated with the genes found in the abstract by the MEDIE system. MEDIE uses the GENA dictionary with entries normalized to Entrez Gene, Swiss-Prot, TrEMBL, Fly-base and several other major databases. To map the names for Gene Entrez IDs to the text we use a fast web service providing cached information from Entrez Gene. The current results from MEDIE are not as good as the GNSuite results, but a new version of MEDIE will be published next year with state-of-the-art results. As more underlying systems are made available as web-services, the online system will be able to process more data on-the-fly. Instead of clicking on one of the article or gene identifiers to annotate the article, the user can type the ID-value with assistance of an auto-completion box with all the potential matching values shown. The gene ID completion-box also shows the total number of articles that each matching gene occurs in.
